# Effects of Nutrition and Exercise Health Behaviors on Predicted Risk of Cardiovascular Disease among Workers with Different Body Mass Index Levels

**DOI:** 10.3390/ijerph110504664

**Published:** 2014-04-29

**Authors:** Jui-Hua Huang, Shu-Ling Huang, Ren-Hau Li, Ling-Hui Wang, Yu-Ling Chen, Feng-Cheng Tang

**Affiliations:** 1 Occupational Health Center, Changhua Christian Hospital, Changhua 500, Taiwan; E-Mails: Juihua55@ms35.hinet.net (J.-H.H.); tokatherinew@gmail.com (L.-H.W.); 396185@cch.org.tw (Y.-L.C.); 2 Department of Community Health, Chia-Yi Christian Hospital, Chiayi 600, Taiwan; 3 Room of Clinical Psychology, Chung-Shan Medical University Hospital, Taichung 402, Taiwan; 4 Department of Psychology, Chung-Shan Medical University, Taichung 402, Taiwan; E-Mail: davidrhlee@yahoo.com.tw; 5 Department of Business Administration, Hwa Hsia Institute of Technology, New Taipei City 235, Taiwan; 6 Department of Leisure Services Management, Chaoyang University of Technology, Taichung 413, Taiwan

**Keywords:** nutrition, exercise, body mass index, CVD, Framingham risk score, health promotion, worker

## Abstract

Workplace health promotion programs should be tailored according to individual needs and efficient intervention. This study aimed to determine the effects of nutrition and exercise health behaviors on predicted risk for cardiovascular disease (CVD) when body mass index (BMI) is considered. In total, 3350 Taiwanese workers were included in this cross-sectional study. A self-reported questionnaire was used to measure their nutrition and exercise behaviors. Data on anthropometric values, biochemical blood determinations, and predicted CVD risk (using the Framingham risk score) were collected. In multiple regression analyses, the nutrition behavior score was independently and negatively associated with CVD risk. Exercise was not significantly associated with the risk. However, the interactive effect of exercise and BMI on CVD risk was evident. When stratified by BMI levels, associations between exercise and CVD risk were statistically significant for ideal weight and overweight subgroups. In conclusion, nutrition behavior plays an important role in predicting the CVD risk. Exercise behavior is also a significant predictor for ideal weight and overweight workers. Notably, for underweight or obese workers, maintaining health-promoting exercise seems insufficient to prevent the CVD. In order to improve workers’ cardiovascular health, more specific health-promoting strategies should be developed to suit the different BMI levels.

## 1. Introduction

Cardiovascular disease (CVD) is a class of disorders affecting the heart and blood vessels, and is considered a significant, universal public health problem [1,2,3]. CVD might result in severe disabilities, particularly among those who survive an atherothrombotic event and it may even cause death. Past studies have indicated that many factors, such as inappropriate diet and a lack of physical activity, can lead to CVD in the general population [4,5,6]. In addition to unhealthy personal lifestyles, poor working conditions, such as long working hours and high occupational stress, put some workers at high risk for CVD [7,8,9,10,11]. Therefore, prevention should be an important issue when promoting workers’ health.

The practice of good nutrition and healthy exercise are common approaches for health promotion in the Taiwanese workplace [12]. Worksite health promotion programs are an effective way to improve workers’ nutritional habits, promote physical activity and reduce obesity [13,14,15]. Several studies have shown that an unhealthy nutritional habit is a significant risk factor linked to CVD [4,16,17]. A prospective cohort study also suggests that a poor dietary pattern can be linked to the increased possibility of hardening of the large arteries [18]. In terms of exercise, regular exercise prevents unhealthy weight gain and reduces the occurrence of CVD [5]. A low level of physical activity has been found to be a predictor of CVD mortality in middle-aged people [6]. Existing studies mainly focus on the association between health-promoting behavior and the onset of CVD. However, little is known about the effects of nutrition and exercise health behaviors on predicting the long-term risk of CVD among workers. The Framingham risk score (FRS) is the most widely used tool to estimate an individual’s global cardiovascular risk [19]. Research has indicated that by using the FRS assessment, a 10-year CVD risk can be calculated, with approximately 75% accuracy, providing insight to the possible benefits of prevention [19,20]. As a result, the present study adopted the FRS as the indicator of predicted CVD risk in workers.

Body mass index (BMI) provides a measure of obesity, and is a predictor of fatness-related health risks [21]. The BMI is also an easily recognized indicator that health-promoting practitioners can use when planning workplace intervention programs. Several studies have shown that people with different BMI levels adopt different healthy lifestyles [22,23]. Individuals who are overweight or obese tend to have poorer diets and a lower participation in physical activities compared to those of normal weight [22,23]. However, the correlation between a health-promoting lifestyle and the BMI level is not linear. Also, obesity itself is pathogenetically related to several clinical and sub-clinical abnormalities that lead to the onset of CVD [24]. Therefore, the effects of nutrition and exercise health behaviors on the long-term CVD risk may be different when workers are stratified by BMI levels. More attention should be paid to the role of BMI levels in these relationships.

This study aimed to bring attention to the prediction of the long-term risk of CVD among workers with different BMI levels, by focusing on a set of nutrition and exercise health-promoting behaviors. The objective of the study was to determine the effects of nutrition and exercise behaviors on workers’ CVD risks, when the BMI is taken into consideration. According to the significant interactive effects of health behaviors and the BMI, in order to promote good health in the workplace, for practical purposes, further analysis would be conducted through stratifying the BMI levels to explore the specific effects of nutrition and/or exercise behaviors on the CVD risk.

## 2. Methods

### 2.1. Study Design

The present study was conducted in 2012, using a cross-sectional research method with convenient sampling. All of the participants volunteered for the study. Personal information and health behaviors were obtained through a self-reported questionnaire. Clinical parameters and bio-data were collected through the identified companies’ annual health screening, which included laboratory testing and non-invasive physical examinations. The study was a component of Taiwan’s Workplace Health Promotion Scheme, and was approved by the Institutional Review Board of the Changhua Christian Hospital (Taiwan).

### 2.2. Assessment of Nutrition and Exercise Health Behaviors

The data of nutrition and exercise health behaviors were obtained by using subscales of the Health Promoting Lifestyle Profile II [25]. The nine questions regarding nutrition behavior were: “choose a low-fat diet”, “limit the use of sugars”, “eat bread, cereal and rice”, “eat fruit”, “eat vegetables”, “eat meat, poultry, fish, dried beans, eggs and nuts”, “eat milk, yogurt or cheese”, “read labels to identify nutrients” and “eat breakfast”. The number of daily servings for each food group was set according to dietary guidelines. The eight items for exercise behavior were: “follow an exercise program”, “vigorous exercise 3 times a week”, “light to moderate physical activity”, “attend leisure-time exercise”, “do stretching 3 times a week”, “get exercise during daily activities”, “check pulse when exercising” and “reach a target heart rate when exercising”. Participants were asked to rate each item on a four-point Likert scale (Never, Sometimes, Often and Routinely). Each subscale’s mean score, ranging from 1 to 4, was calculated from the subscale’s score totals and divided by the number of response items. A higher score indicated a greater level of participation in health-promoting behavior. In this survey, the subscales of nutrition and exercise behaviors showed acceptable internal consistency, with Cronbach’s alphas of 0.85 and 0.78, respectively.

### 2.3. Anthropometric Measurements

Anthropometric measurements included body height and weight. The body mass index (BMI) was calculated with weight divided by the height squared ((kg)/(m^2^)). The body mass index, according to the Health Promotion Administration, Taiwan Ministry of Health and Welfare, was categorized into four levels: underweight (BMI < 18.5), ideal weight (18.5 ≤ BMI < 24.0), overweight (24.0 ≤ BMI < 27.0) and obese (BMI ≥ 27.0).

### 2.4. Assessment of the CVD Risk

In this study the FRS measured the 10-year risk prediction for CVD [19,26]. The FRS total was calculated by adding scores taken from six clinical and laboratory parameters: age, gender, smoking habits, total cholesterol, HDL and systolic blood pressure. Cutoff points on the FRS list were used to differentiate between three estimation levels for the 10-year risk: low risk (<10%), intermediate risk (10%–20%) and high risk (>20%) [19,26].

### 2.5. Statistical Analysis

All statistical procedures were performed using SPSS 17.0 statistical software (SPSS Inc., Chicago, IL, USA) where a *p*-value of less than 0.05 was considered statistically significant. Before analysis, any data that had more than 25% of the questions incomplete were excluded to ensure the credibility of results. For categorical variables in the contingency table, data were presented in numbers (*n*) and percentages (%), and were analyzed by the Chi-square test. For continuous variables, data were presented in the mean ± SD, and mean comparisons were analyzed by a 2-tailed *t*-test (2 groups) or a one-way ANOVA (>2 groups). The *post-hoc* Scheffe method was then used to determine the pairwise differences between the group means when the ANOVA results were significant. Associations of nutrition and exercise behaviors with FRS totals were examined in a multiple regression analysis, after adjustment for gender and age; the interactive effects between health behaviors and BMI on the risk were also simultaneously investigated in this model. The multiple regression analysis models were extended once the significant interactive effects occurred. That is, subsequent regression analyses by strata of BMI levels were carried out to explore specific effects of nutrition and/or exercise health behaviors on the predicted CVD risk. The FRS values were log transformed before analysis because of the data’s skewed distribution. The regression analysis outcomes are presented in unstandardized coefficients (B) and standardized coefficients (β), at a 95% Confidence Interval (CI) for B.

## 3. Results

### 3.1. Participants’ Baseline Characteristics

For the study, a total of 3362 workers, aged 20 years or over, were recruited from three companies. However some of them were unable to provide the necessary information on personal data, nutrition and exercise health behaviors, or a physical examination, resulting in a final number of 3350 participants, with an average age of 47.8 ± 8.4. Using their information, characteristics regarding nutrition and exercise behaviors, BMI, laboratory parameters and smoking were summarized in Table 1. Male workers had a significantly higher FRS total, BMI, and systolic blood pressure than the females did, and there were indications of gender differences in health lifestyles, including exercise, nutrition and smoking (*p* < 0.001). Male workers had lower nutrition health behavior scores than did the females, but the exercise health behavior scores were higher for males than for females. In addition, Pearson correlation analysis was conducted to examine any correlations between age with nutrition and exercise behaviors, BMI and the FRS totals. The results (not shown in the table) showed that age was positively correlated with nutrition and exercise behaviors, and FRS (*p* < 0.001). Age was also marginally and positively correlated with BMI (*p* = 0.072). Since relations of age and gender with almost all reported correlates were statistically significant, age and gender were then considered as control variables in subsequent regression analysis models.

**Table 1:** Descriptive statistics of the participants’ personal characteristics by gender.

Characteristic ^‡^	Total	Male	Female	*p*^ †^
	(*n* = 3350)	(*n* = 2569)	(*n* = 781)	
Age (year)	47.8 ± 8.4	48.3 ± 8.8	46.4 ± 7.0	<0.001
Nutrition health behaviors score	2.5 ± 0.4	2.5 ± 0.4	2.6 ± 0.4	<0.001
Exercise health behaviors score	2.0 ± 0.6	2.1 ± 0.6	2.0 ± 0.5	0.001
Body mass index (kg/m^2^)	24.1 ± 3.4	24.4 ± 3.3	23.0 ± 3.5	<0.001
Framingham risk score (%)	4.7 ± 5.0	6.1 ± 5.0	0.5 ± 0.8	<0.001
Total cholesterol (mg/dL)	194.3 ± 34.1	194.1 ± 34.1	195.0 ± 34.0	0.552
Systolic blood pressure (mmHg)	125.5 ± 15.4	127.7 ± 14.3	118.1 ± 16.3	<0.001
Smoking				
With	528 (17.5)	520 (22.6)	8 (1.1)	<0.001
Without	2486 (82.5)	1778 (77.4)	708 (98.9)	

^† ^*p*-value less than 0.05 was considered statistically significant. ^‡^ Continuous data are presented in mean ± SD. Categorical data are presented in number (*n*) and percent (%).

**Table 2:** Comparison of FRS levels on BMI using chi-square test, and on health behaviors using one-way ANOVAs and post-hoc comparisons.

	Framingham risk score levels			
	Low risk (<10%) (*n* = 2470)	Moderate risk (10%–20%) (*n* = 461)	High risk (>20%) (*n* = 29)	*p*^ †^
BMI (kg/m^2^) ^‡^				<0.001
BMI < 18.5 (underweight)	82 (94.3)	5 (5.7)	-	
18.5 ≤ BMI < 24 (ideal weight)	1241 (88.2)	157 (11.2)	9 (0.6)	
24 ≤ BMI < 27 (overweight)	774 (79.8)	189 (19.5)	7 (0.7)	
BMI ≥ 27 (obesity)	373 (75.2)	110 (22.2)	13 (2.6)	
Health behaviors *				
Nutrition health behavior score ^§^	2.6 ± 0.4	2.5 ± 0.4	2.6 ± 0.5	0.012
Exercise health behavior score	2.0 ± 0.6	2.1 ± 0.6	2.1 ± 0.6	0.157

^†^ *p*-value less than 0.05 was considered statistically significant. ^‡^ Categorical data are presented in number (*n*) and percent (%). Continuous data are presented in mean ± SD. ^§^ indicates significant difference in nutrition health behavior score between low risk and moderate risk groups by Scheffe’s multiple comparisons test (*p* = 0.015), but other two comparisons (low *vs.* high, and moderate *vs.* high) are not significant. * Both nutrition and exercise scores ranged from 1 (never) to 4 (routinely).

### 3.2. FRS Levels by BMI and Health Behaviors

As shown in Table 2, the FRS levels are significantly associated with the BMI levels and nutrition behavior score. There are trends towards increasing percentage of the moderate/high CVD risk as BMI level increases. The obese group is at a greater CVD risk than are other groups. Besides, when the associations between FRS and health behaviors are analyzed by ANOVA, in regard to exercise behavior, there were no statistically significant differences among the three groups of FRS (*p* = 0.157). However, there is an overall significant difference in nutrition behavior among three FRS levels (*p* = 0.012). Following Scheffe’s *post-hoc* comparisons, the low risk group had a higher score of nutrition behavior than those in the moderate risk group.

### 3.3. Relationship between Health Behaviors and Log FRS by BMI Levels

Multiple linear regression analysis was conducted to examine the effects of nutrition and exercise health behaviors on the log-transformed FRS, using gender and age as the adjusted variables (Table 3). The nutrition behavior score was inversely correlated with the CVD risk (*p* < 0.001), but its interaction with BMI on the risk was not statistically significant. 

**Table 3:** Multiple regression models predicting Framingham risk score in relation to nutrition, exercise and BMI.

Variable	Log Framingham risk score (%)			
	B	β	*p*	95% CI for B
Gender	1.029	0.606	<0.001	(0.997, 1.062)
Age	0.050	0.544	<0.001	(0.048, 0.052)
Health behaviors				
Nutrition health behavior	−0.073	−0.044	<0.001	(−0.107, −0.038)
Exercise health behavior	−0.018	−0.014	0.176	(−0.044, 0.008)
BMI (kg/m^2^)	0.024	0.110	<0.001	(0.020, 0.028)
Nutrition health behavior × BMI	−0.005	−0.009	0.376	(−0.015, 0.006)
Exercise health behavior × BMI	0.010	0.026	0.012	(0.002, 0.018)
BMI levels				
Underweight				
Gender	1.000	0.680	<0.001	(0.847, 1.153)
Age	0.053	0.687	<0.001	(0.044, 0.061)
Exercise health behavior score	−0.086	−0.057	0.294	(−0.247, 0.076)
Ideal weight				
Gender	1.068	0.652	<0.001	(1.024, 0.051)
Age	0.049	0.539	<0.001	(0.046, 0.051)
Exercise health behavior score	−0.056	−0.044	0.001	(−0.090, −0.022)
Overweight				
Gender	0.990	0.569	<0.001	(0.931, 1.048)
Age	0.052	0.574	<0.001	(0.049, 0.056)
Exercise health behavior score	−0.040	−0.034	0.046	(−0.079, 0.000)
Obesity				
Gender	1.081	0.598	<0.001	(0.992, 1.169)
Age	0.047	0.546	<0.001	(0.043, 0.051)
Exercise health behavior score	0.004	0.003	0.910	(−0.058, 0.065)

All outcomes of the regression analysis are presented in unstandardized coefficients (B), standardized coefficients (â), at a 95% Confidence Interval (CI) for B. Unstandardized coefficient (B) represents the effect of one unit change in the explanatory variable on FRS. For example, in the overweight group, as the exercise score increases one unit, FRS would decrease by 0.040%. Likewise, standardized coefficient (â) represents the effect of one standard deviation change in the explanatory variable on the standardized score of FRS. For example, in the overweight group, as the exercise score increases by one standard deviation, FRS would decrease by 0.034 standard deviation.

On the other hand, the exercise score was not significantly associated with the CVD risk in the model. However, a significant interaction was found between exercise and BMI for predicting CVD risk (*p* = 0.012). The main effects of exercise on predicting CVD risk, when stratified by BMI levels, were then executed in the next step of regression analyses. Specifically, results from the ideal weight and overweight groups showed that the exercise score was inversely correlated with the log FRS (*p* = 0.001 and *p* = 0.046).

## 4. Discussion

This study aimed to explore the effects of nutrition and exercise on the predicted CVD risk, when an individual’s BMI was taken into consideration. The study’s data show that obese workers had a higher CVD risk, than those of other BMI levels. Furthermore, nutrition behavior was independently and negatively associated with the CVD risk, whereas exercise was not significantly predictive of the risk. A strong association between exercise and the CVD risk, however, was found in ideal weight and overweight subgroups, when individuals were stratified by their BMI levels.

Good nutrition is one of the most important factors in the prevention and management of CVD [3,4]. The nutrition behavior score was inversely correlated with the FRS totals (Table 3). These findings were similar to a previous study which had indicated that a group with a high CVD risk (FRS > 20%) had poorer dietary intake than those in the low risk (FRS < 10%) group [27]. In general, a healthy diet is high in fruits, vegetables, grain products, lean meats and fiber, and low in fat [28,29]. It also decreases the probability of obesity [4]. Individuals need to try to reduce their obesity risk so that they may reduce the risk of a number of chronic diseases, including diabetes, hypertension and CVD [24]. On the other hand, an unhealthy nutritional habit is a CVD risk factor [4,16]. A prospective study suggested that an unhealthy dietary pattern causes metabolism syndromes [16], which in turn may raise the risk of developing diabetes and CVD, and increase cardiovascular morbidity and mortality [17,30]. A prospective cohort study also suggested that a nutritionally poor dietary pattern was related to the increased chance of hardening of the large arteries [18]. This study’s findings also suggest that an appropriate nutrition behavior may predict a lower long-term risk of CVD among workers. 

The workers should be encouraged to implement proper nutrition behaviors, such as taking fiber-rich diets, lowering sodium and fat in foods, and not skipping meals, to prevent CVD. In regard to exercising, the study found that the independent effect of exercise behavior on the CVD risk was not evident (Table 3). However, when stratified by BMI levels, the exercise behavior score was inversely associated with a log-transformed FRS for the ideal weight and overweight workers (about 80% of the participants). The health benefits of exercise depend on the extent of the activities, including intensity, frequency and duration [31]. An inactive lifestyle is a high-risk factor for CVD. Previous studies found that although a low level of physical activity was a predictor for CVD mortality, adequate exercise may generally improve fitness and prevent or remedy CVD [5,6]. A regular and effective exercise behavior is beneficial for cardiovascular health by preventing unhealthy weight gain and positively affecting blood glucose and lipids [5,32]. Unexpectedly, any evident effects of healthy exercise on their CVD risk were not found with obese participants. A possible reason for this non-significant association may be attributed to other factors, for instance, chronic low-grade inflammation, which can lead directly to the development of CVD [33]. Therefore, for obese workers, maintaining health-promoting exercise behavior may not be sufficient to reduce their long-term CVD risk. This study observed the different effects of healthy exercising on the CVD risk in workers with different BMI levels. In order to improve their workers’ health, the workplace’s health promotion practitioners should develop more specific exercise strategies to suit the different BMI levels.

In the present study, male workers had a significantly higher CVD risk than did the females. These disparities in CVD risk may be due to lifestyle differences [34,35,36]. The study’s data showed that males had poorer nutritional habits compared to the females (Table 1), a finding that was consistent with a previous study [37]. Dietary patterns are linked to health-related behaviors that result in chronic diseases, including obesity and CVD [4,27]. In terms of exercise, males scored significantly higher than females, which is consistent with another study’s findings [38]. Previous studies showed that adequate exercise might, generally, reduce the occurrence of CVD [5]. However, the prevalence of smoking was significantly higher among the male workers. Smoking is one of the parameters for calculating the FRS [26]. The difference in smoking habits may contribute to the difference in the CVD risk between male and female workers. This notable gender gap can also be found in Taiwan’s national smoking survey for adults [39]. In addition, this study revealed gender disparities in health behaviors, echoing findings from in other studies [35,36]. The gender difference in healthy lifestyle, therefore, should be considered when tailoring health promotion programs. For the prevention of CVD, male workers might benefit from reducing smoking and a strengthening of their nutrition behavior; for females, an establishment of healthy exercise might be advisable.

Findings in this study bring about some policy implications in workplace health promotion. Nutrition health-promoting behavior has been verified in the study to be beneficial for improving cardiovascular health. However, the mean score of nutrition behavior for all participants in the study is only at a moderate level and still has room for improvement. More effective strategies in helping workers maintain a good dietary habit are recommended. Additionally, workplace health promotion practitioner should consider the BMI levels of workers when designing those health-promoting exercise programs aiming for a reduction of individual’s long-term CVD risk. Our research suggests that exercise might not be sufficient in reducing the occurrence of CVD for the underweight and obese groups of workers. Further research to identify the key factors in protecting these groups from cardiovascular harm may be needed. Although this study has provided some insights into the effects of exercise and nutrition health behaviors on predicted risk of CVD among workers, it has several limitations. Firstly, it was a cross-sectional study and could not establish the causal direction of the links between nutrition and exercise behaviors and the predicted CVD risk. Secondly, the analyses of the nutrition and exercise behaviors relied heavily on data gathered from a self-reported questionnaire. Overestimation or underestimation may occur when participants assess their own health behaviors. To overcome these shortcomings, it is recommended that more longitudinal research be conducted with more objective tools for assessing health behaviors.

## 5. Conclusions

This study suggests that nutrition behavior is a key component in the prediction of the CVD risk for workers. Furthermore, the effects of exercise behavior on the predicted CVD risks were found to vary among workers with different BMI levels. Therefore, it is recommended that workplace health promotion practitioners should develop more specific strategies to target workers with different BMI levels. For underweight and obese workers, maintaining a healthy lifestyle, in terms of exercise only, may prove to be insufficient in reducing the CVD risk. It is suggested that the factors associated with the CVD risks for these workers need further investigation.

## References

[1] Nabel E.G. (2003). Cardiovascular disease. N. Engl. J. Med..

[2] World Health Organization Cardiovascular Diseases (CVDs) Fact Sheet. http://www.who.int/mediacentre/factsheets/fs317/en/index.html.

[3] Ueshima H., Sekikawa A., Miura K., Turin T.C., Takashima N., Kita Y., Watanabe M., Kadota A., Okuda N., Kadowaki T. (2008). Cardiovascular disease and risk factors in Asia: A selected review. Circulation.

[4] Abdel-Megeid F.Y., Abdelkarem H.M., El-Fetouh A.M. (2011). Unhealthy nutritional habits in university students are a risk factor for cardiovascular diseases. Saudi Med. J..

[5] Haskell W.L., Lee I.M., Pate R.R., Powell K.E., Blair S.N., Franklin B.A., Macera C.A., Heath G.W., Thompson P.D., Bauman A. (2007). Physical activity and public health: Updated recommendation for adults from the American College of Sports Medicine and the American Heart Association. Med. Sci. Sports Exerc..

[6] Barengo N.C., Hu G., Lakka T.A., Pekkarinen H., Nissinen A., Tuomilehto J. (2004). Low physical activity as a predictor for total and cardiovascular disease mortality in middle-aged men and women in Finland. Eur. Heart J..

[7] Yang H., Schnall P.L., Jauregui M., Su C.T., Baker D. (2006). Work hours and self-reported hypertension among working people in California. Hypertension.

[8] Park J., Kim Y., Cho Y., Woo K.H., Chung H.K., Iwasaki K., Oka T., Sasaki T., Hisanaga N. (2001). Regular overtime and cardiovascular functions. Ind. Health.

[9] Kivimäki M., Batty G.D., Hamer M., Ferrie J.E., Vahtera J., Virtanen M., Marmot M.G., Singh-Manoux A., Shipley M.J. (2011). Using additional information on working hours to predict coronary heart disease: A cohort study. Ann. Intern. Med..

[10] Belkic K.L., Landsbergis P.A., Schnall P.L., Baker D. (2004). Is job strain a major source of cardiovascular disease risk?. Scand. J. Work Environ. Heath.

[11] Su C.T., Yang H.J., Lin C.F., Tsai M.C., Shieh Y.H., Chin W.T. (2001). Arterial blood pressure and blood lipids as cardiovascular risk factors and occupational stress in Taiwan. Int. J. Cardiol..

[12] Chiu C.J., Liu G.L. (2003). Cognition, attitude and demands regarding health promotion of employees and their healthy lifestyles-current situation and related factors. Health Promot. Health Educ. J..

[13] Ni Mhurchu C., Aston L.M., Jebb S.A. (2010). Effects of worksite health promotion interventions on employee diets: A systematic review. BMC Public Health.

[14] Buller D., Buller M.K., Larkey L., Sennott-Miller L., Taren D., Aickin M., Wentzel T.M., Morrill C. (2000). Implementing a 5-a-day peer health educator program for public sector labor and trades employees. Health Educ. Behav..

[15] Stokes G.C., Henley N.S., Herget C. (2006). Creating a culture of wellness in workplaces. N. C. Med. J..

[16] Lutsey P.L., Steffen L.M., Stevens J. (2008). Dietary intake and the development of the metabolic syndrome—The Atherosclerosis Risk in Communities study. Circulation.

[17] Haffner S.M., Valdez R.A., Hazuda H.P., Mitchell B.D., Morales P.A., Stern M.P. (1992). Prospective analysis of the insulin-resistance syndrome (syndrome X). Diabetes.

[18] Kesse-Guyot E., Vergnaud A.C., Fezeu L., Zureik M., Blacher J., Péneau S., Hercberg S., Galan P., Czernichow S. (2010). Associations between dietary patterns and arterial stiffness, carotid artery intima-media thickness and atherosclerosis. Eur. J. Cardiovasc. Prev. Rehabil..

[19] Kang C.S., Chern C.I., Lin M.S., Lai Z.Y., Chang N.C., Chiou C.S., Lee T.M. (2006). New advance in risk prognostication for coronary event. J. Intern. Med. Taiwan.

[20] Sheridan S., Pignone M., Mulrow C. (2003). Framingham-based tools to calculate the global risk of coronary heart disease: A systematic review of tools for clinicians. J. Gen. Intern. Med..

[21] Mooney S.J., Baecker A., Rundle A.G. (2013). Comparison of anthropometric and body composition measures as predictors of components of the metabolic syndrome in a clinical setting. Obes. Res. Clin. Pract..

[22] Wolongevicz D.M., Zhu L., Pencina M.J., Kimokoti R.W., Newby P.K., D’Agostino R.B., Millen B.E. (2010). Diet quality and obesity in women: The Framingham Nutrition Studies. Br. J. Nutr..

[23] Liebman M., Pelican S., Moore S.A., Holmes B., Wardlaw M.K., Melcher L.M., Liddil A.C., Paul L.C., Dunnagan T., Haynes G.W. (2003). Dietary intake, eating behavior, and physical activity-related determinants of high body mass index in rural communities in Wyoming, Montana, and Idaho. Int. J. Obes. Relat. Metab. Disord..

[24] Abate N. (2000). Obesity and cardiovascular disease. Pathogenetic role of the metabolic syndrome and therapeutic implications. J. Diabetes Complicat..

[25] Walker S.N., Sechrist K.R., Pender N.J. Health Promoting Lifestyle Profile II. http://wwwunmcedu/nursing/Health_Promoting_Lifestyle_Profile_IIhtm.

[26] Ford E.S., Giles W.H., Mokdad A.H. (2004). The distribution of 10-year risk for coronary heart disease among US adults—Findings from the National Health and Nutrition Examination Survey III. J. Am. Coll. Cardiol..

[27] Sohn C., Kim J., Bae W. (2012). The framingham risk score, diet, and inflammatory markers in Korean men with metabolic syndrome. Nutr. Res. Pract..

[28] McGuire S. (2011). U.S. Department of Agriculture and U.S. Department of Health and Human Services, *Dietary Guidelines for Americans, 2010*. 7th Edition, Washington, DC: U.S. Government Printing Office, January 2011. Adv. Nutr..

[29] Taiwan Department of Health (2011). Dietary Guidelines for Taiwan. http://www.fda.gov.tw/news.aspx?newssn=7828&key_year=0&keyword=&classifysn=4&unitsn=1.

[30] Isomaa B., Almgren P., Tuomi T., Forsen B., Lahti K., Nissen M., Taskinen M.R., Groop L. (2001). Cardiovascular morbidity and mortality associated with the metabolic syndrome. Diabetes Care.

[31] U.S. Department of Health and Human Services: 2008 Physical Activity Guidelines for Americans. http://www.health.gov/paguidelines/guidelines/default.aspx.

[32] Colberg S.R., Albright A.L., Blissmer B.J., Braun B., Chasan-Taber L., Fernhall B., Regensteiner J.G., Rubin R.R., Sigal R.J. (2010). Exercise and type 2 diabetes: American college of sports medicine and the american diabetes association: Joint position statement. Exercise and type 2 diabetes. Med. Sci. Sports Exerc..

[33] Wang Z., Nakayama T. (2010). Inflammation, a link between obesity and cardiovascular disease. Mediat. Inflamm..

[34] Millen B.E., Quatromoni P.A., Copenhafer D.L., Demissie S., O’Horo C.E., D’Agostino R.B. (2001). Validation of a dietary pattern approach for evaluating nutritional risk: The framingham nutrition studies. J. Am. Diet. Assoc..

[35] Pender N.J., Walker S.N., Sechrist K.R., Frank-Stromborg M. (1990). Predicting health-promoting lifestyles in the workplace. Nurs. Res..

[36] Lusk S.L., Kerr M.J., Ronis D.L. (1995). Health-promoting lifestyles of blue-collar, skilled trade, and white-collar workers. Nurs. Res..

[37] Lin W., Hang C.M., Yang H.C., Hung M.H. (2011). 2005–2008 Nutrition and health survey in Taiwan: The nutrition knowledge, attitude and behavior of 19–64 year old adults. Asia. Pac. J. Clin. Nutr..

[38] Ammouri A.A. (2008). Demographic differences in health promoting lifestyle of adult Jordanians. J. Med. J..

[39] Bureau of Health Promotion, Department of Health, R.O.C. (Taiwan): Bureau of Health Promotion (2010). Taiwan Tobacco Control Annual Report 2010. http://health99.doh.gov.tw/media/public/pdf/21661.pdf.

